# Proceedings of the Chicago Medical Society

**Published:** 1869-02-15

**Authors:** 


### PROCEEDINGS OF SOCIETIES.

### *Proceedings of the Chicago Medical Society.*

### "SPONTANEOUS" DISLOCATION OF THE LENS INTO THE ANTERIOR CHAMBER.

Dr. Holmes presented a crystalline lens, which he had extracted from the right eye of a young man, 22 years of age, a student in the Rush Medical College. In his infancy there was a marked failure in the development of the osseous system which produced considerable distortion of the ancle and wrist joints and of the sternum. As early as in the sixth month of his age, his parents had observed that he was extremely near sighted. From this time to his nineteenth year, he was very myopic, with almost absolutely no power of accommodation. In reading he was obliged to hold his book a distance of only three inches.

At the end of this period he observed that he could see distant objects with the left eye over the lens and near ones through it; on stooping he could see only very near objects. The lens had become slightly dislocated downward. Two years ago the left lens fell into the anterior chamber, while the patient was in the simple act of stooping. A surgeon replaced the lens behind the iris, by an operation with a needle. Severe inflammation with hydrophthalmus and final atrophy of the globe followed.

About a year ago there was a partial displacement of the right lens downward with the effects on vision as above described. On the 7th of October last, while stooping forward in his chair, the lens fell into the anterior chamber. With the lens in this position he could read the finest diamond print at the distance of two inches behind the cornea. The lens resembled a beautiful globule of air.

In consultation with Prof. Gunn, I was decided to extract the lens at once, before irritation supervened. There was reason to apprehend, with the progressive atrophy of the zonula of Zinn, that there was also dissolution of the vitreous humor.

The lens was removed through an incision made with Graefe's knife, in the lower portion of the cornea, encroaching upon the limbus conjunctivæ, but not so far upon the sclerotic as in the modified peripheral extraction. As the knife traversed the anterior chamber, the lens was crowded into the upper portion of the anterior chamber, repressing the upper portion of the iris into the vitreous humor. As the aqueous humor was evacuated, the lower free border of the iris rose in front of the knife and was excised. The lens passed out spontaneously without the slightest loss of vitreous humor. The edges of the wound were in exact coaptation, as the lids were placed in position and the compressive bandages applied.

In ten days the patient was about his room with good vision, having suffered scarcely any pain from the operation. In a few days there appeared a minute transparent staphyloma in the apex of the cicatrix, just below the transparent borders of the cornea. The iris was not involved, as the globe was quite tense; it was thought best to make a minute opening through the staphyloma. Although no pressure was exerted upon the globe, and the patient suffered no pain to cause spasm of the muscles, a large quantity of perfectly limpid fluid, like aqueous humor, was evacuated. The quantity was such that there could be no doubt that a portion of it was the dissolved vitreous humor. Although the tension of the eye became very much reduced, the wound healed, and the eye was soon restored to its normal firmness, *without impairing vision.*

The patient returned home to an adjoining State, able to read or write without the use of a lens. Six weeks after the extraction he wrote a letter, as he stated, with as much ease as ever before in his life. That same night while on the cars he awoke almost totally blind. He discovered on palpation no loss of the intraoccular contents. He came at once to Chicago in great pain. In less than twenty-four hours there was much œdema of the conjunctiva, and lids with quite a copious muco-purulent discharge, as if the patient was suffering from an attack of uncomplicated acute conjunctivitis. A delicate greyish membrane could be seen floating behind the iris in the inner and lower portion of the globe, which was, undoubtedly, a detached portion of the retina. No ophthalmoscopic examination was made on account of the severe pain. Violent inflammation continued nearly two weeks. The patient soon returned home, blind, with scarcely a hope that any subsequent operation could restore his vision. An examination of the refraction of the eye was deferred after the extraction of the lens, in expectation of the patient's returning to the city when the eye had perfectly recovered from the effects of the operation. Dr. Holmes also presented another crystalline lens which he had recently removed from the anterior chamber. The patient, 43 years of age, had received a blow on the left eye 25 years ago which nearly destroyed the vision. For twenty years there was a small white body lying *in* the pupil, a part of which projected into the anterior chamber. Five years ago, while in the act of lifting a great weight, the entire lens fell into the anterior chamber. During this period he had suffered frequent attacks of pain and irritation. For the purpose of relieving irritation with scarcely a hope of improving vision, the lens was removed, through an opening in the lower portion of the anterior chamber. The wound healed readily without any irritation. The lens was much atrophied, very hard and white in appearance, composed entirely of calcareous matter.

### AMPUTATION AT THE SHOULDER JOINT.

Dr. Wanzer exhibited the fragments of the upper portion of the humerus which he had amputated at the shoulder joint, of a German, 51 years of age, whose arm had been crushed in a machine. The patient made a good recovery.

Dr. Bogue exhibited an ovum which had evidently been blighted, in consequence of hæmorrhage between the amnion and chorion. The clot of blood and the placenta could be easily traced. Neither the fœtus nor cord could be found. Dr. Bogue exhibited for Dr. Hutchinson a beautiful specimen of the neck of a tapeworm.

